# Programmed cell death: the pathways to severe COVID-19?

**DOI:** 10.1042/BCJ20210602

**Published:** 2022-03-04

**Authors:** Stefanie M. Bader, James P. Cooney, Marc Pellegrini, Marcel Doerflinger

**Affiliations:** 1 The Walter and Eliza Hall Institute of Medical Research, Parkville, VIC 3052, Australia; 2 The Department of Medical Biology, University of Melbourne, Parkville, VIC 3010, Australia

**Keywords:** apoptosis, Covid-19, inflammation, necroptosis, pyroptosis, therapeutics

## Abstract

Two years after the emergence of SARS-CoV-2, our understanding of COVID-19 disease pathogenesis is still incomplete. Despite unprecedented global collaborative scientific efforts and rapid vaccine development, an uneven vaccine roll-out and the emergence of novel variants of concern such as omicron underscore the critical importance of identifying the mechanisms that contribute to this disease. Overt inflammation and cell death have been proposed to be central drivers of severe pathology in COVID-19 patients and their pathways and molecular components therefore present promising targets for host-directed therapeutics. In our review, we summarize the current knowledge on the role and impact of diverse programmed cell death (PCD) pathways on COVID-19 disease. We dissect the complex connection of cell death and inflammatory signaling at the cellular and molecular level and identify a number of critical questions that remain to be addressed. We provide rationale for targeting of cell death as potential COVID-19 treatment and provide an overview of current therapeutics that could potentially enter clinical trials in the near future.

## Introduction

On New Years eve 2019, media reports emerged ∼27 cases of viral pneumonia in the central city of Wuhan, China [1]. Within the following two weeks, Chinese health officials linked the outbreak to a Coronavirus with similar taxonomy to SARS-CoV, the virus that caused Severe Acute Respiratory Syndrome (SARS) and led to almost 800 fatalities in 2002–2003. The characteristic disease symptoms evoked by the novel virus were similar to SARS, including pneumonia, fever, cough, and in severe cases dyspnea and lung infiltration [2].

Since then, unprecedented global scientific collaborative efforts have been made to curb the spread of the disease, resulting in the fastest vaccine development ever achieved [3], as well as recent approvals of virus- and host-directed therapeutics. However, despite all the rapid progress, our understanding of COVID-19 disease pathogenesis, almost 2 years after the emergence of SARS-CoV-2, is far from complete and there is a continuing urgency to dissect the contributors to this disease which has been responsible for over 5 million deaths [4]. Although rapid vaccine roll out across many countries has been tremendously effective in mitigating morbidity and mortality, its distribution has not been universal. The most recently identified variant of concern, B.1.1.529 (Omicron), contains 32 mutations in the spike protein [5], and it has capacity to reinfect people who have recovered from previous infection with other variants. It is likely that two doses of our current vaccines will be less effective in preventing infection and disease caused by omicron [6]. This highlights a critical need for continued efforts to identify the molecular mechanisms underlying COVID-19 disease to improve current treatment options. Inflammation and cell death have been proposed to be central drivers of disease pathology in COVID-19 patients (as reviewed in [7,8]). These reports suggest that targeting host cell death pathways may have therapeutic value in mitigation severe COVID-19.

Here we review the literature during the first two years of the SARS-CoV-2 pandemic and focus on the role and impact of programmed cell death (PCD) on COVID-19. We will dissect potential mechanisms for disease at the cellular and molecular level and identify critical gaps in our understanding of severe COVID-19 disease pathogenesis. We will attempt to identify potential therapeutic avenues for COVID-19 based on our present understanding of host cell death pathways and their contribution to disease.

## Coronaviruses, SARS-CoV-2 and COVID-19 disease pathology

Coronaviruses are a group of related RNA viruses that cause respiratory and intestinal infections in animals and humans [9]. Severity of disease can range from mild, in case of common cold viruses, to more lethal varieties caused by severe acute respiratory syndrome coronavirus (SARS-CoV) [4], Middle East respiratory syndrome–related coronavirus (MERS-CoV) [10], and SARS-CoV-2. Coronaviruses are enveloped viruses with long positive-sense, non-segmented single-stranded RNA genomes ranging from 27 to 33 kb in size. The coronavirus genomes comprise a variable number (from 6 to 11) of open reading frames (ORFs), which encode for non-structural proteins (nsps) [11], and four major structural proteins: the spike (S), envelope (E), membrane (M) and nucleocapsid (N) proteins [12].

SARS-CoV-2 enters cells through engagement of angiotensin converting enzyme 2 (ACE-2) and the subsequent events can be mediated by two distinct ways: (a) Endocytosis and the cutting of the SARS-COV-2 spike protein by cathepsin B, L and calpain in endosomes; [13,14] (b) non-endosomal pathways, where SARS-CoV-2 is primed by the serin proteases TMPRSS2 and furin [15,16]. Despite using the same entry mechanism as SARS-CoV, SARS-CoV-2 initially infects epithelial cells of the upper respiratory tract [17], which is believed to cause the higher levels of viral shedding [18] and transmissibility that is characteristic to this strain [15,19].

In the majority of cases, COVID-19 manifests as mild cold-like symptoms, but severe disease with complications occurs in ∼15% of patients. Severity of disease correlates with age and co-morbidities, with older men (>60 years old) being more likely to require hospitalization [20]. Severe disease is characterized by respiratory failure and systemic inflammatory reactions with overt cytokine release. In critically ill patients a cytokine storm causes lung injury characteristic of acute respiratory distress syndrome (ARDS), which is considered the main cause of death in COVID-19 patients. Predictors of disease severity include ferritin, TNF-α, IL-8, IL-1β and IL-6 [21–28], suggesting that hyperinflammation is driving COVID-19 severity and death [25]. Increased monocytes, low-density neutrophils and eosinophils in lungs of patients are also correlated with worse illness [29].

The exact mechanism of SARS-CoV-2 viral spread through the respiratory tract, kinetics of infection and cytokine release are still under investigation. Multiple studies using other coronaviruses [8,30,31] have attempted to help fill the gaps in our knowledge of COVID-19 disease pathogenesis. However, although SARS-CoV-2 is in the same Betacoronavirus clade as MERS-CoV and SARS-CoV, there are notable differences between their whole genome and amino acid sequences, with SARS-CoV-2 being closer related to bat coronaviruses than other viruses associated with outbreaks in humans [32,33]. Wu and colleagues compared SARS-CoV-2 to related viruses and showed only the proteins nsp7, nsp13, envelope, matrix and accessory proteins p6 and 8b remained fully conserved. Furthermore, several significant differences arose in SARS-CoV-2 compared with its relative SARS-CoV: the 8a protein is completely absent from SARS-CoV-2, while 8b and 3b proteins differ in length between both viruses [33], indicating that comparisons between their functionalities and interactions with host (programmed cell death) pathways need to be assessed carefully.

SARS-CoV-2 infection starts in the upper-airways via the naso-oral cavity, likely infecting and replicating within epithelial cells. During the initial phase of infection, the virus spreads down the lower respiratory tract, infecting pneumocytes and triggering the innate immune response [34,35]. During viral lung infections, death of infected cells triggers the release of PAMP/DAMPs, thereby activating and recruiting alveolar and monocyte-derived macrophages and neutrophils. These infiltrated and activated immune cells produce pro-inflammatory cytokines resulting in more cell death, PAM release and further innate immune cell recruitment. During SARS-CoV-2 infection, activation of the Nod-like receptor family pyrin domain-containing 3 (NLRP3) inflammasome pathway is known to contribute further to the pro-inflammatory environment with the release of IL-1β, IL-18 and DAMPs [36–38]. Excessive activation of NLRP3 leads to high DAMPs release, pyroptosis, activation of macrophages, neutrophil infiltration, cytokine production (IL-1β, IL-2, IL-6, IL-17, TNF-α, G-CSF, GM-CSF, IFN-γ, CXCL10, CCL2, and CCL3), cell death and fibrosis, which are all key features characteristic of severe COVID-19 [38].

**Figure 1.**
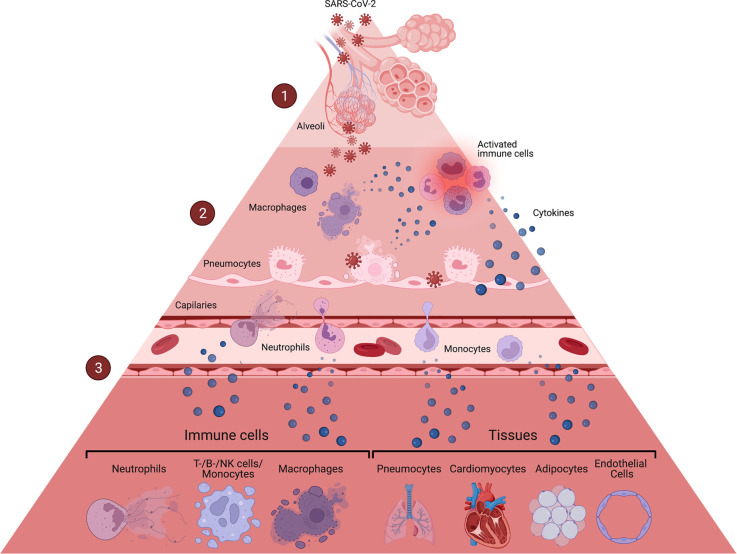
COVID-19 disease leads to death of multiple cell types. (**1**) SARS-CoV-2 infects the upper-airways via the naso-oral cavity, sensitizing epithelial cells to cell death [39,40]. The virus quickly spreads to alveoli in the lower respiratory tract, infecting pneumocytes and triggering the innate immune response [33,34]. (**2**) Infected pneumocytes undergo pyroptosis [22], releasing PAMP/DAMPs and cytokines, which leads to the activation and recruitment of alveolar and monocyte-derived macrophages and neutrophils. SARS-CoV-2 infected macrophages were associated with markers of proptosis and necroptosis [39,41,42], two lytic forms of cell death, which further contribute to the release of pro-inflammatory cytokines. The recruited neutrophils were also associated with necroptosis, but are thought to mainly undergo NETosis in the context of COVID-19, which can be detrimental to surrounding cells and tissues [39,43–46]. (**3**) Activated and dying macrophages and neutrophils produce pro-inflammatory cytokines resulting in more cell death of neighboring cells, PAM release and further innate immune cell recruitment, initiating a cascade of cytokines, leading to cell death in multiple tissues and cell types.

While multiple questions regarding host pathogen interactions during COVID-19 remain open, the consensus is that the pro-inflammatory milieu generated by the immune reaction to SARS-CoV-2 replication and the link between inflammation and cell death are the major contributors to COVID-19 pathogenesis (Figure 1).

## Programmed cell death during SARS-CoV-2 infection

The association of severe COVID-19 with a dysregulated hyperinflammatory immune response has led to a huge interest in targeting inflammatory mediators and their key activation pathways as COVID-19 therapeutics. However, Programmed Cell Death (PCD) pathways are often upstream of inflammatory processes, potentially playing a central role in mediating severe disease caused by SARS-CoV-2 infection. The main PCD pathways, apoptosis, pyroptosis and necroptosis can cooperate and create redundancy in offering defence against intracellular infection [47]. Additionally, they can initiate and coordinate innate and adaptive immune responses and inflammation (as reviewed in [48]). PCD can be advantageous to the host in removing infected cells and promoting immunity, but it can also cause disease through collateral tissue damage if it is not highly regulated. The role of PCD and its contribution to mitigating or promoting disease is likely dependent on which programmed cell death pathways predominate and this in term is dependent on the pathogen and sequence of events immediately following infection.

Cytokines are key drivers of inflammatory processes, acting synergistically to define the fate of different cell types [49]. In BMDMs, a combination of cytokines associated with severe COVID-19 (IL-6, IL-18, IFN-γ, IL-15, TNF-α, IL-1α, IL-1β, and IL-2) was shown to robustly induce cell death [26], indicating that the hyper-inflammatory state created by the immune system in response to SARS-CoV-2 inevitably leads to activation of PCD pathways. As discussed throughout this review, this is supported by emerging evidence identifying the activation of key mediators of the host inflammasome and cell death machinery in a range of immune and other cells (Table 1) during SARS-CoV-2 infection that fuel pathology and severe disease outcomes.

**Table 1:** Forms of cell death reported to be implicated in COVID-19

Cell type	Cell death pathway	References
T-Cells	Apoptosis (extrinsic/p53)	[27,50–52]
NK and B-cells	Apoptosis	[53]
pDCs	Apoptosis	[41]
Macrophages	Pyroptosis/Necroptosis	[39,42,54]
Monocytes	Apoptosis/Pyroptosis	[53,55,56]
Pneumocyte	Pyroptosis	[23]
Cardiomyocytes	Apoptosis	[57]
Adipocytes	Necroptosis/Apoptosis	[58]
Endothelial cells	Apoptosis	[40]
Epithelial cells	Apoptosis/Necroptosis	[54,59]
Platelets	Apoptosis/Necroptosis	[43]
Neutrophils	NETosis/Necroptosis	[44–46,54,60]
Pancreatic islets	Necroptosis	[61]
Adrenal glands	Necroptosis	[43]

In the following chapters, we will summarize our current understanding on how different host programmed cell death mechanisms shape inflammation and impact tissue damage and COVID-19 disease pathogenesis.

## Death of neutrophils: NETosis and ROS

Neutrophils are critical for the first line of innate immune responses to infection and are activated through pathogen recognition receptors (PRRs), which induce phagocytosis. Engulfed pathogens are exposed to granules containing proteases, defensins, antimicrobial peptides or reactive oxygen species (ROS) [62]. Neutrophil extracellular trap (NET) formation (NETosis), a form of PCD, is considered a further effector function of neutrophils, neutralizing intruders through the release of chromatin structures containing histones and antimicrobial proteins [63].

Severe COVID-19 is marked by dysregulated and excessive neutrophil function (reviewed in [62]). Patients with severe disease display a higher neutrophil to lymphocyte ratio (NLR) compared with mild cases, and studies have proposed this as a predictive marker of death [20,64]. Serum of severely ill COVID-19 patients showed increased markers associated with NETosis, such as cell-free DNA [45], myeloperoxidase-DNA [44,45] and citrullinated histone H3 [45]. Veras and colleagues also found increased NET formation in tracheal aspirates and lung autopsies from COVID-19 patients [44], indicating that NETosis occurs during COVID-19 and might contribute towards severe disease.

Active, intracellular infection of neutrophils is likely a mechanism responsible for NET formation. In vitro, SARS-CoV-2 infection was able to induce an increase in DNA release to a level similar to that of PMA stimulation, a compound used to experimentally induce NETosis in neutrophils [46]. Furthermore, neutrophils were shown to express ACE-2 and neutralization of the SARS-CoV-2 entry receptors with anti-hACE2 antibody or camostat, an inhibitor of the serine protease TMPRSS2, reduced NET formation *in vitro* [44]. Interestingly, sera from COVID-19 patients triggered NET release from neutrophils of healthy controls [45], suggesting that neutrophils might also undergo NETosis through indirect mechanisms, such as cytokine activation, rather than intracellular infection. The exact roles of intracellular infection and pro-inflammatory cytokines in NET formation during COVID-19 are yet to be defined. The studies to date have used *in vitro* infection models and it remains unclear if neutrophils constitute a major infection reservoir in Human disease.

NETosis has long been associated with worsening pulmonary disease during respiratory infections in people with cystic fibrosis. Neutrophilic death is thought to increase mucus viscosity and obstruction, contributing to a decline of pulmonary function [65]. Pathological NET formation is also associated with the occlusion of arteries and vessels in degenerative cardiovascular disease [66], which could be an additional mechanism contributing to the coagulopathy associated with severe COVID-19. Additionally, NET formation can be detrimental to surrounding cells, as shown by increased death of lung epithelial cells after the release of NETs by SARS-CoV-2–activated neutrophils [44]. This indicates that inhibition of NETosis might have therapeutic value during COVID-19 to alleviate symptoms of severe disease. Therapeutic options targeting NETosis during COVID-19 have been discussed previously [67].

Neutrophils have also been associated with excessive production of Reactive Oxygen Species (ROS) during COVID-19 [46,68]. It is well established that viral infections are associated with increased levels of ROS [69–71], and during COVID-19 the high neutrophil to lymphocyte ratio observed in severely ill patients is positively associated with increased ROS. This is thought to exacerbate the host immunopathological response, resulting in even more severe disease [68]. While low levels of ROS are essential for normal physiological function, its accumulation causes damage to proteins, nucleic acids, lipids, membranes and organelles, ultimately leading to cell death [72]. ROS can trigger cell death by multiple pathways, leading to necrotic or apoptotic death [72].

Collectively, while further studies are required to clarify the role of NETosis during COVID-19, neutrophil dysregulation, characterized by oxidative stress and aberrant NET formation appears to play a role in severe disease [73]. Additionally, NETs promote production of type I interferons [74] and other cytokines [75] that can exacerbate the pro-inflammatory milieu characteristic of COVID-19.

## Cell death mediated by interferon responses

Interferon (IFN) responses are a major defence mechanism against viral infections [76–80]. Initially, IFNs were mainly associated with antiviral defence through the inhibition of viral replication [80], but over the past years IFN-signaling has also been associated with transcriptional responses that are linked to multiple cell death pathways [26,76–79,81–83]. Among the main cytokines produced during SARS-CoV-2 infection are IFN-γ and TNF-α, the combination of which can induce inflammatory cell death [26]. This indicates that IFN signaling might act synergistically with TNF to create a pro-inflammatory environment, leading to cell death.

There are three distinct IFN families, which all exert antiviral and immune modulatory functions. The type I IFN family encodes IFNα and IFNβ subtypes [84], which are produced by a wide range of immune cells and if dysregulated can cause cell death, inflammation and immunological host responses, ultimately leading to tissue injury [85]. The type II IFN family consists of IFNγ, which is predominantly produced by T cells and NK cells to stimulate antigen-specific adaptive immunity and innate immunity. Finally, the type III IFN family includes IFNλ [84], which is induced at early stages of viral infection to suppress the initial viral spread without activating inflammation [77,84].

Several studies have found correlations between interferon responses and severe COVID-19 [21,25,29,78]. Increased IFN-α [78], IFN-γ-inducible protein 1 [21,25] and plasma IFNγ levels have been found in patients with more severe forms of the disease [21,29], indicating that a dysregulated IFN response is detrimental at least in a proportion of patients. As always, this data needs to be interpreted with caution as these responses may be consequence rather than the cause of severe disease. Kim et al. showed an association between type I interferon and a hyper-inflammatory state linked to severe COVID-19. This hyperinflammatory state was mediated by IRF-1 [78]. IRF-1 regulates ZBP1 in the NLRP3 inflammasome, leading to cell death during influenza infection [86], but a direct link between IRF-1 and cell death during SARS-CoV-2 infection has not yet been established. A longitudinal study reports that during the first week of symptoms, IFNλ levels increase in patients with severe disease and remains elevated in later phases [29], while others show decreased IFNγ levels in ICU patients, compared with patients with less severe disease [78]. Higher IFNα levels were sustained in patients with severe disease, while in moderate cases IFNα decreased with time [29].

IFN responses vary across different cell types during SARS-CoV-2 infection. Single-cell transcriptional analysis of peripheral blood mononuclear cells (PBMCs) in COVID-19 patients showed up-regulation of interferon stimulated genes (ISGs) in T, B, NK, and DC cell subsets, including *X-linked inhibitor of apoptosis* (*XIAP*)*-associated factor 1* (XAF1) [27]. XAF1 exerts a pro-apoptotic function through a positive feedback loop with p53 [87] and interferon regulatory factor-1 (IRF-1), a transcription factor associated with type I IFN responses [77], demonstrating an interconnection between IFN signaling and cell death pathways. Consistently, increased expression of IRF1, TP53 and CASP3 were seen in COVID-19 patients compared with controls [78]. A single-cell transcriptional study performed in PBMCs of COVID-19 patients further suggests the involvement of IFNα and IFNγ in promoting severe disease via activation of STAT1/IRF3 in CD4^+^ and CD8^+^ T cells, naive T cells, and DCs [27]. Single-cell RNA sequencing of antigen presenting cell subsets (APCs), a central source of IFNs, showed critical defects in the response to type I IFN in severe COVID-19 patients, suggesting that dysfunctional IFN production, receptor expression and downstream ISG responses [41] correlate with poor disease outcomes. Furthermore, mutations in IFN genes and autoantibodies against type I IFNs in blood have been shown to affect response to SARS-CoV-2 [88], highlighting how subtle differences in host defence can lead to different cytokine profiles and disease outcomes.

Collectively, the role of IFN signaling during host-pathogen response in COVID-19 is complex and somewhat contradictory (reviewed in [89]). Zanoni highlights the fact that there are studies demonstrating a protective role of IFN during SARS-CoV-2 infection, while others propose IFNs as drivers of disease severity [89]. These contradictory results may be related to the timing of measurements during disease as well as the organ/site analysed. Overall, studies suggest that type I IFN responses are dysfunctional in severe COVID-19 [24,88,90–96] and correlate with TNF- and IL-1β-driven inflammation in patients with severe disease [92]. The location, timing and duration of IFN exposure, as well as the host genetics, may conspire to promote disease progression. This dysregulated response likely causes substantial tissue damage through cell death, both through type I IFN, which can kill via a variety of pathways (reviewed in [85]) and via IFNγ, which in synergy with TNF-α appears to play a detrimental role by inducing cell death during COVID-19 [26]. The exact role of IFNs and their ability to induce cell death during COVID-19 is presently unclear.

## Apoptosis

Apoptotic cell death is triggered by a death ligand, such as tumor necrosis factor (TNF), which binds its cognate receptor in the case of extrinsic apoptosis, or by diverse stress stimuli which activate the mitochondrial intrinsic death pathway. The intrinsic pathway involves two critical proteins, BCL-2-associated protein (BAX) and BCL-2 homologous antagonist killer (BAK). These proteins cause mitochondrial outer membrane permeabilization (MOMP), which results in activation of the initiator Caspase-9 leading to apoptosis. Intracellular BH3-only proteins can trigger death via the mitochondria, through inhibition of pro-survival B-cell lymphoma-2 (BCL-2)-regulated proteins [97]. In contrast, extrinsic apoptosis is triggered when death ligands bind their cognate death-receptors leading to the activation of Caspase-8. The process is regulated by cellular inhibitor of apoptosis proteins (cIAPs). These proteins prevent activation of Caspase-8 and instead cause the activation of NFκB, leading to the production of inflammatory cytokines. Both intrinsic and extrinsic cell death pathways lead to the activation of executioner Caspases (Caspases-3 and -7), to generate a cascade of proteolytic events ultimately leading to cell death [98]. Apoptotic death destroys intracellular content in a coordinated manner. The generation of membrane-enclosed apoptotic vesicles containing degraded cellular components and dead intracellular pathogens prevents the release of viable microorganisms [99,100].

Coronaviruses are known to engage apoptotic pathways via diverse mechanisms. The SARS-CoV accessory proteins ORFs 3a, 6, 7a, 7b, 8a and 9b, as well as the structural proteins M and N, were associated with apoptosis (reviewed in [8]), but their relevance during *in vivo* infection still needs to be clarified. Several studies addressing the role of viral proteins in cell death show they can lead to apoptosis through a variety of signaling events. While SARS-CoV ORF7a was linked to Caspase-3 activation, likely through activation of p38 Mitogen Activated Protein Kinase (MAPK) [101], SARS-CoV-2 ORF3a induced apoptosis in HEK293T, HepG2, and VeroE6 cells via Caspase-8, -9 and BID activation [102]. SARS-CoV-2 ORF3a is proposed to require membrane association to exert its apoptotic activity, while SARS-CoV ORF3a and ORF6 can trigger apoptosis independently of membrane association [102]. SARS-CoV ORF6 was shown to induce apoptosis via Caspase-3 and the activation of the ER-stress pathway [103,104]. ORF6 is conserved between SARS and SARS-CoV-2 [33], suggesting that it might play a role in COVID-19. In addition to SARS-CoV-2 viral proteins ORF, M and N, the spike protein subunit 1 (S1) also triggers apoptosis in human adipocytes, evidenced by increased cleavage of caspases 8 and 3 and TUNEL-positive cells [58].

As discussed above, differences between genome and amino acid sequences as well as differences in the clinical manifestations of disease in MERS-CoV, SARS-CoV and SARS-CoV-2, indicate that these viruses interact with the host in different ways to cause disease [32] and cell death. A study comparing apoptosis pathways in all 3 coronaviruses showed how protein kinase R-like endoplasmic reticulum kinase (PERK) signaling modulates apoptosis during MERS-CoV infection *in vivo*, but not during SARS-CoV and SARS-CoV-2 infections [105]. PERK can trigger apoptosis and autophagy [106]. During active infection, the large number of viral proteins produced by infected cells can cause ER stress through different pathways, which all converge on the activation of intrinsic apoptosis if homeostasis cannot be rescued [76,107]. Chu et al. [105] showed that regardless of the mechanism through which apoptosis was initiated, its inhibition ameliorated disease in mouse models of infection. The Caspase-9 inhibitor z-LEHD-fmk reduced lung damage in the K18-hACE2 transgenic SARS-CoV-2 mouse model, but the study did not measure viral burdens in the lungs. z-LEHD-fmk treatment of mice also reduced TNF-α, IL-6 and IP10 levels, indicating that intrinsic apoptosis signaling, characterized by Caspase-9 activation plays a role in cytokine release and contributes to inflammation during SARS-CoV-2 infection *in vivo* [105]. Nevertheless, it cannot be ruled out that z-LEHD-fmk is also targeting other Caspases, such as Caspase-8 or -1, thereby inhibiting cytokine processing or other forms of PCD. Another comparison between SARS-CoV-2, MERS-CoV and SARS-CoV was performed by interrogating gene expression datasets of transcriptional signatures in infected human lung epithelial cells. Changes in genes related to apoptosis [59] were identified, suggesting that the epithelial cells, which are the primary infection site in early stages of disease, might be the first compartment sensitized to death upon infection.

A hallmark of patients with severe COVID-19 is lymphopenia, which correlates with poor clinical outcomes [108–110]. Although T cells are particularly affected [53,111,112], especially CD8^+^ [29,112] and CD4^+^ and T cells [29], B- [113] and NK cells are also decreased [41,113]. Retention of lymphocytes in lymphoid tissue and in sites of viral replication might contribute to systemic lymphopenia [114], but lung inflammation was also shown to be a be a contributing factor [20] that could promote lymphocytic death during COVID-19. Apoptosis and P53 signaling were suggested to be involved in the loss of lymphocytes [51–53] and pDCs [41] during SARS-CoV-2 infection. PBMC transcriptome analysis [51], whole-blood [52] and single cell RNA sequencing [41] were utilized to find correlations between p53 activation and viral infection in COVID-19 patients. P53 is known to be involved in virus-mediated apoptosis and it has been implicated as a direct transcriptional target of type I interferons [115], which further implicates a role for this tumor suppressor gene in antiviral immunity and cell death during COVID-19.

It has become apparent that cell death pathways are highly interconnected within cells, providing multiple avenues for the induction of death [47]. T cell numbers in blood were shown to negatively correlate with levels of IL-6, IL-10, and TNF-α [50], indicating that apoptosis of T cells might also be occurring as a result of the extrinsic pathway triggered by activation of TNFR1. Zhu et al. [27] observed elevated expression of TNFSF10 (TRAIL) and its receptor TNFRSF10 in T cells from COVID-19 patients and proposed a potential increase in TNF- and FAS-induced T cell apoptosis during SARS-CoV-2 infection. Interestingly, TNF-α induced apoptosis of lymphocytes was shown to increase with age [116,117], and may potentially link extrinsic apoptosis with the increased risk of severe COVID-19 in older people.

It is unclear whether SARS-CoV-2 directly infects B and T cells [118], indicating that death of lymphocytes may be caused by indirect mechanisms, such as overall increased cytokine levels, rather than viral infection exerting direct cytopathic effects on these cells. A study using RNAscope to detect SARS-CoV-2 RNA in patient-derived lung samples showed active infection of pneumocytes, macrophages and endothelial cells, but not infiltrating lymphocytes. This may provide evidence that the main cause of lymphocyte death might be through cytokine activity or other stress induced mechanisms and not the result of intracellular viral replication [23].

A study using scRNAseq analysis of antigen presenting cells (APCs) suggests that a decrease in circulating pDCs, which has been observed during COVID-19 [119], might be related to expression of pro-apoptotic molecules [41]. Similar to lymphocytes, there is evidence that APC death is not due to direct virus-induced killing, as *in vitro* SARS-CoV-2 stimulation of pDCs leads to improved pDC survival [120].

Apoptosis during COVID-19 has been mainly linked to immune cell depletion, but it has also been suggested to play a role in coagulopathy, via death of endothelial cells [121], as well as in platelets [43] and cardiomyocytes [57]. Platelets were reported to die after internalization and digestion of SARS-CoV-2 virions [43]. This process might contribute to thrombocytopenia, as well as dysregulated immunity and thrombosis during COVID-19. Cardiomyocytes were also shown to be infected by SARS-CoV-2, leading to cell death and reduced contractility [57].

Overall, accumulating evidence suggest that the apoptotic cell death that occurs in multiple immune and non-immune cell types during COVID-19 plays a role in disease severity, making it a potential therapeutic target. However, the highly dynamic and complex pro-inflammatory cytokine milieu throughout different stages of SARS-CoV-2 infection and COVID-19 disease [21–29,122] likely elicits distinct responses and activation of different PCD pathways across different cell populations in a time and tissue specific manner. As an example, pDCs from COVID-19 patients seem to have increased TNF signaling, while the same pathway is decreased in monocytes [41]. This underscores the complexity in interpreting *in vitro*, *ex vivo* and *in vivo* data and the implications of these data sets for disease severity and therapeutic interventions.

## Pyroptosis and inflammasome activation

Pyroptosis is a form of necrotic and inflammatory PCD induced by the inflammatory Caspases-1/-4/-5 or -11 [123]. Caspase activation during pyroptosis is regulated by inflammasomes in response to infections or specific host derived proteins and crystals [124,125]. The inflammasome sensors NLRP3, AIM2 and Pyrin require the adaptor protein ASC to form a functional Caspase-1-containing inflammasome complex, while NLRC4 and NLRP1b inflammasomes can directly bind to Caspase-1 without ASC. Caspase-1 mediates processing of IL-1β and IL-18 into their active forms, as well as cleavage of the pore-forming protein gasdermin D (GSDMD) that induces pyroptotic cell death and the release of pro-inflammatory cytokines [123,126]. Mature IL-1β induces the recruitment of innate immune cells and modulates the activity of adaptive immune cells. In contrast mature IL-18 is involved in the production of IFN-γ and stimulation of Th1 and Th2 responses [127,128].

Inflammasomes need to be primed to induce cell death and cytokine release [128]. Engagement of toll-like or cytokine receptors such as IL-1R and TNFR trigger NFkB signaling and transcription of inflammasome complex subunits. A second signal is required to activate the inflammasome complex, which can be triggered by K^+^ efflux, Ca^2+^ signaling, reactive oxygen species (ROS), mitochondrial dysfunction, and lysosomal rupture [128]. NLRP3 activation and pyroptosis have been linked to expression of specific SARS-CoV-2 proteins. ORF3a was shown to act as a K^+^ channel (Chen et al. 2019) and to disrupt mitochondrial integrity, leading to intracellular accumulation and NLRP3 inflammasome activation [129]. Orf8b and the E envelope can also act as ion channels, potentially activating NLRP3 by K^+^ efflux [55,130–132].

The NLRP3 inflammasome has been linked to systemic inflammatory responses in a range of infectious and inflammatory diseases [133–136] and there is extensive evidence suggesting that the NLRP3 inflammasome is activated during SARS-CoV-2 infection [36–38,137]. NLRP3 activation has been proposed as an indicator of COVID-19 disease severity [37,137], predicting the release of pro-inflammatory cytokines that lead to dysregulated immune responses and tissue damage [129]. Many studies focus on the role of cytokines linked to NLRP3 activation, rather than on the specific role of pyroptotic cell death. Since IL-1β can be released without pyroptosis [138], the two processes, cytokine release and cell death, need to be studied independently during COVID-19 pathogenesis.

Multiple studies analysing blood of COVID-19 patients have found evidence of pyroptotic cell death [55,56,139]. Pre-prints by independent groups showed increased plasma biomarkers of pyroptosis, including IL-1RA, IL-18, LDH and GSDMD [55] and cleaved Caspase-1 [139] which correlated with the development of severe disease. Both studies show evidence of NLRP3 inflammasome activation and Caspase-1 cleavage in blood immune cells, indicating pyroptotic cell death is occurring upon SARS-CoV-2 infection. Rodrigues et al. [36] observed the release of IL-1β and IL-18 in PBMCs isolated from COVID-19 patients, while Junqueira and colleagues showed that circulating monocytes are infected with SARS-CoV-2 and undergo pyroptosis [55]. This is consistent with results on human primary monocytes demonstrating that SARS-CoV-2 activates the inflammasome and Caspase-1, increasing IL-1ß levels and leading to GSDMD pore formation [56].

Inflammasome-induced cytokines were also correlated with disease severity [29]. Blockage of IL-1ß receptors was shown to reduced Caspase-1 activation and cell death, indicating a positive feedback loop in which pro-inflammatory cytokines cause more inflammation due to increased pyroptosis [56], leading to increased disease severity. There are regulatory mechanisms to counteract the over-production of pro-inflammatory cytokines occurring during severe COVID-19. The negative regulator of IL-1 signaling, IL-1 receptor antagonist (IL-1Ra) [140], was increased in patients with severe disease [29], suggesting that negative feedback mechanisms are triggered in attempt to ameliorate the cytokine storm.

Even though several studies suggest Caspase-1 activation and pyroptosis during SARS-CoV-2 infection, the response seems to be cell type specific. *In vitro*, the SARS-CoV-2 ORF8b protein was shown to trigger NLRP3 activation in macrophages [39,42]. The relevance of NLRP3 inflammasome activation was shown to differ among the different types of myeloid cells in severe COVID-19 disease [37]. Single-cell RNA-sequencing of different APC populations showed increased IL-1ß levels in patients with severe disease compared with moderate cases and uninfected controls [41], implicating specific immune cell subtypes in the release of pro-inflammatory cytokines.

There is also increasing evidence of pyroptosis in non-immune cells. In lung tissues of diseased COVID-19 patients, GSDMD positive pneumocytes were found using immunohistochemistry, indicating that SARS-CoV-2 infection triggers pyroptosis in human lung tissue [23]. A study focusing on adipose tissue suggests that cultured adipocytes treated with SARS-CoV-2 spike protein subunit 1 undergo pyroptosis, via activation of NLRP3, ASC, active Caspase-1, cleavage of GSDMD and secretion of IL-1β [58]. The same study also observed cleavage of Caspases-8 and -3, indicating that multiple death pathways can be triggered in the same cell upon SARS-CoV-2 infection.

Additionally, activation of non-canonical pyroptosis mediators during SARS-CoV-2 infection has been reported. Comparison of plasma from patients with severe and mild COVID-19 showed that in severe disease, expression of quantitative trait loci of GSDME was elevated [55]. Cleavage of GSDME and Caspases-3/-7/-9 was also shown in a study analysing syncytia formation using cells expressing ACE-2 and SARS-CoV-2 spike. Treatment with ZVAD and gene ablation of Caspase-9, but not Caspase-8, blocked cleavage of GSDME, suggesting that Caspase-9 might mediate death of syncytia through GSDME activation [141]. GSDME was also shown to be cleaved in PBMCs of healthy donors after treatment with a combination of TNF-α and IFNγ, two cytokines linked to COVID-19 severity [26], further indicating a potential role of GSDME as a backup mechanism for cell death during SARS-CoV-2 infection.

Interestingly, one study reports inhibition of pyroptosis in human monocytes upon SARS-CoV-2 infection [142]. Ma and colleagues propose that the viral nucleocapsid binds to the GSDMD linker region, inhibiting cleavage by Caspase-1, but in contrast, the same study also shows IL-1ß being expressed in non-classical monocytes in patients with mild to moderate disease [142].

Inflammasome activation and pyroptosis have been the focus of extensive research due to their connection with cytokine processing and release of IL-1β, which has been shown to play a critical role in the development of severe COVID-19 [24,27,91,92,143–150]. Accumulating evidence showing pyroptosis of monocytes, macrophages, pneumocytes and adipocytes suggests that this pro-inflammatory form of cell death is a major contributor to the cytokine dysregulation and disease severity during COVID-19. However, the vast majority of studies either rely on cell lines and *in vitro* studies or correlative datasets generated from human patient derived samples and thus fail to show clear evidence for the role of pyroptosis independent of cytokine release in COVID-19 pathogenesis.

## Necroptosis

Necroptosis is another lytic form of PCD that releases DAMPs, causing immune activation, cytokine release and inflammation [151]. Necroptotic cell death can be induced downstream of death receptor signaling, including TNF receptor 1 (TNFR1), via cytoplasmic DNA sensor Z-DNA binding protein 1 (ZBP1) [152,153] and downstream of Toll-like receptors and activation of TIR domain-containing adapter-inducing interferon-β (TRIF) [154,155]. Downstream of TNFR1, receptor interacting serine/threonine kinases 1 (RIPK1) regulates signaling to cause activation of NF-kB, apoptosis or necroptosis [156]. Necroptosis occurs when RIPK1 promotes the activation of RIPK3. This only occurs in the absence of functional Caspase-8 [156]. Activated RIPK3 then phosphorylates the pseudokinase mixed lineage kinase domain-like (MLKL), leading to its activation oligomerization and translocation to the cell membrane where it forms pores and eventually leads to cell death [157,158].

Interestingly, MLKL protein expression was shown to be regulated by type I and II interferon signaling [159,160]. This ‘necroptotic priming’ during inflammation may be relevant in the pathogenesis of COVID-19 because of aberrant cytokine storms linked to disease. Importantly, given the apparent role of inflammasome activation and IL1-β secretion in the development of severe COVID-19 disease, it is noteworthy that MLKL was shown to trigger the NLRP3 inflammasome and IL1-β secretion through a cell intrinsic pyroptotic and GSDMD independent mechanism [161,162].

Increased levels of circulating RIPK3 were found in patients with severe COVID-19 [163] and several reports have identified hallmarks of necroptosis signaling across a spectrum of host cells during SARS-CoV-2 infection, including lung epithelial cells [54], neutrophils [54,60], macrophages [54], platelets [43], adipocytes [58], pancreatic islets [61] and in adrenal glands [43]. Using *in vitro* techniques, SARS-CoV-2 infection was shown to up-regulate phosphorylated MLKL (p-MLKL) in lung epithelial cell lines, which was dependent on viral replication and rescued by RIPK3 inhibition [164]. Differentiated adipocytes stimulated with viral proteins showed a tendency towards increased RIPK1 phosphorylation and robust RIPK3 and MLKL phosphorylation [58]. Koupenova at al report up-regulated necroptosis signaling using RNASeq analysis as well as increased p-MLKL levels in platelets infected with SARS-CoV-2 [43]. Junqueira at al identified increased expression of RIPK3, ZBP1 and Caspase-8 in blood samples of severe COVID-19 cases using expression quantitative trait loci (eQTLs) gene profiling [55].

Interestingly, ORF3a of SARS-CoV was shown to bind RIPK3 and elicit a multimodal necrotic cell death that was dependent of NLRP3 and Caspase-1, but independent of MLKL [165]. Using MHV (mouse hepatitis virus) as model for coronavirus induced inflammatory cell death *in vitro*, Zheng et al. report MLKL activation and necroptosis to be induced as back up mechanism when NLRP3 mediated pyroptosis via Caspases-1/11 and GSDMD was disabled, and that the necroptotic death observed was critically dependent on Caspase-8 and RIPK3 [166]. In a mouse model using TNF and IFN-γ administration as surrogate model for the cytokine storms induced by SARS-CoV-2, RIPK3 deficiency failed to protect against cell death, whereas deletion of both RIPK3 and Caspase-8 provided substantial protection against the cell death induced by cytokine treatment [26].

RIPK1 driven neutrophil necroptosis was identified in critically ill COVID-19 patients [60], and RIPK1 activation was detected in lungs of COVID-19 patients which was at least in part a result of infiltration of RIPK1+ macrophages and neutrophils [54]. Inhibition of RIPK1 using Nec1s was shown to reduce SARS-CoV2 replication *in vitro* [22] as well as in human lung organoids and in infected CAG-hAce2 mice [54]. Interestingly, while neither the active/phosphorylated forms of RIPK3 nor MLKL were detectable in lungs of COVID-19 patients in this study, high gene expression levels of MLKL were identified in lung and airway epithelial cells, macrophages and neutrophils [54]. This provides evidence that MLKL activation in COVID-19 may not occur downstream of activated RIPK1–RIPK3 to promote necroptosis, but rather by alternative RIPK3-independent inflammatory responses such as via IFNγ as previously reported in inflammatory arthritis [167].

As the vast majority of these data are correlative, based solely on *in vitro* findings or obtained using non-SARS-CoV-2 models, the exact role of necroptotic cell death vs. inflammatory signaling evoked by RIPK1 and/or RIPK3 remains to be established in COVID-19. It is tempting to speculate that this cell death modality simply occurs as consequence of collateral damage.

## The emerging evidence of interconnected cell death pathways and their contribution to the pro-inflammatory state in severe COVID-19

Cell death mechanisms, especially lytic forms such as necroptosis and pyroptosis, are coupled to the release of cytokines. Fundamental discoveries over the last decade have established that PCD is controlled with high plasticity by Caspases-1 and -8 [40, Gurung, 2014 #293]. Deciphering their role and regulation during COVID-19 might increase our understanding of how the detrimental cytokine cascades are initiated, sustained and eventually controlled. During infections, multiple pathways can be engaged creating a redundancy in the induction of cell death but importantly, different forms of cell death may have vastly different consequences *in vivo* [168–172]. It is interesting to speculate that disease outcomes may be associated with the type of cell death that is most dominate in the host.

Traditionally, activation of the NLRP3 inflammasone is regarded to be the main inducer of pathogenic pro-inflammatory cytokines IL-1β, IL-18 and IL-6 during COVID-19, but it has become clear that multiple non-conical pathways can contribute and indeed become the dominate inducers of IL-1β. Necroptotic signaling can lead to release of mature IL-1β, independent of GSDMD [161,162,173]. The disruption of the cell membrane through MLKL leads to efflux of potassium ions, NLRP3 inflammasome activation and subsequent maturation of IL-1β, which can be released during necroptosis. Li and colleagues have shown evidence of this during SARS-CoV-2 infection, since both inhibition of RIPK3 and translocation of MLKL reduced IL-1β maturation in infected lung cells [164]. Even though multiple mechanisms can result in IL1 release, the form of cell death triggering cytokine release dictates the nature of the inflammatory mechanism [174]. IL-1β can also be processed and released without cell death, through exosome formation via ASC and NLRP3 activation, independently of Caspase-1 [138], suggesting that IL-1β alone cannot be used as a marker of cell death.

GSDMD, traditionally linked to pyroptotic death, was shown to cooperate in enhancing membrane disruption during other forms of cells death. During apoptosis, GSDMD has been linked to the disruption of mitochondrial membranes and the release of cytochrome C [175] while in NETosis, neutrophil elastase cleaves GSDMD to facilitate granule and plasma membrane permeabilization [176]. Interestingly, NETosis was also shown to activate pyroptosis, through induction of NLRP3 [177], suggesting that during COVID-19 NETosing neutrophils might also contribute to IL1-β and IL-18 secretion.

Caspase-8 was primarily identified and characterized as the initiator Caspase of extrinsic apoptosis. It transduces signals from death receptors to drive potent cellular responses including inflammation, proliferation and/or death [178]. Active Caspase-8 inhibits necroptosis mediated by RIPK3 and MLKL, which causes embryonic lethality in mice lacking the *Casp-8* gene. More recently, Caspase-8 has been identified as a master regulator of an extensive cross-talk between apoptosis and pyroptosis [180, Doerflinger, 2020 #95, Mascarenhas, 2017 #110], even though it was traditionally linked to apoptosis only [179]. When Caspase-1 is absent, Caspase-8 drives inflammasome activation and IL-1β maturation [180, Shenderov, 2014 #289, Bossaller, 2012 #290, Gringhuis, 2012 #291, Antonopoulos, 2013 #292, Gurung, 2014 #293, Man, 2013 #294, Gurung, 2014 #293], through direct cleavage of pro-IL-1β, at the same site targeted by Caspase-1 [180]. Additionally, both Caspases-8 and -9 were recently shown to be upstream of GSDME cleavage, leading to the release of pro-inflammatory cytokines and causing pyroptotic cell death through non-canonical pathways [181,182].

Multiple studies suggest the involvement of Caspase-8 in pathogenesis of COVID-19. Proteomic profiling revealed increased levels of Caspase-8 in blood of both ICU and non-ICU patients [183], while in lung epithelial cells, SARS-CoV-2 infection was shown to induce Caspase-8 and BID cleavage [164]. During SARS-CoV-2 infection, inhibition of Caspase-8 resulted in decreased IL-1β secretion, but not less viral replication [164], suggesting the involvement of Caspase-8 in pro-inflammatory signaling during COVID-19. Karki and colleagues show that concurrent deletion of Caspase-8 and RIPK3 ameliorated cell death and disease outcome in mice treated with TNF-and IFN-γ, supposedly mimicking the detrimental cytokine signaling in COVID-19 disease [26].

Many PCD pathways appear to be activated in COVID-19 but deciphering which are dominant, which are redundant and which are causal of disease is complex. Many, if not all of these pathways are also inextricably linked to the production of cytokines characteristic of severe COVID-19 disease. This adds further to the complexity and confusion of whether the cell death pathways are the cause or effect of cytokine storms. Genetic and pharmacological targeting of cell death pathways may provide some clarity but these studies need to be performed using true *in vivo* models of SARS-CoV-2 infection. It is clear that COVID-19 is not due to disease in a single cell type nor through induction of a single cellular pathway. This complexity can only be dissected *in vivo*. Pharmaceutical targeting of one single pathway will likely not be sufficient to fully ameliorate the pro-inflammatory pathology, since lack of one pathway can usually be compensated by others. Even though a limited number of cytokines appear to be key drivers of severe COVID-19, the plethora of mechanisms and functional redundancies promoting their release and cellular responses need to be assessed in great detail. Additionally, the contribution of individual or combined cell death pathways in specific cell types (i.e. pyroptosis in innate immune cells and apoptosis in epithelial cells) and their downstream effects on disease progression and on innate and adaptive immune responses warrant further investigation.

## Therapeutics

Host directed therapies, together with effective antivirals, will be important weapons in the fight against COVID-19, particularly in those for whom an effective vaccination strategy is not possible (i.e. the immunocompromised). Given the relatively short time frame since the emergence of SARS-CoV-2 (∼2 years at the time of publication), a remarkable number of repurposed host-directed therapies have progressed to the clinic to treat severe COVID-19, including cytokine antagonists, recombinant cytokines, glucocorticoids, and cell-based therapies (reviewed [184]). These strategies show varying degrees of success [185–187].

It is now well understood that host PCD and inflammation are intimately linked, providing rationale to target not only inflammatory effectors, but upstream cell death mediators, an approach thus-far largely unexplored in COVID-19. Pharmacologically curtailing inflammatory cell death or promoting immunologically silent cell death processes may be powerful approaches to ameliorate disease. Many therapeutic drugs that target host PCD pathways have been progressed into the clinic for the treatment of diverse cancers and inflammatory diseases and these can be readily repurposed to treat severe COVID-19 (Table 2).

**Table 2:** Clinical stage therapeutics that target host cell death pathways for potential repurposing into COVID-19

	Drug	Host target	Development stage for COVID19	Development stage for other indications	Ref/clinical tiral identifier
Apoptosis	Navitoclax	BCL2, BCL-xL, BCL-w	None	Phase 3	-
	Venetoclax	BCL2	None	Phase 4	-
	S64315 (MIK665)	MCL1	None	Phase 2	-
	LCL-161	CIAP1/2	None	Phase 2	-
	Xevinapant	CIAP1/2	None	Phase 3	-
	Emricasan	Caspases	None	Phase 2	-
Necroptosis	GSK2982772	RIPK1	None	Phase 2	-
	GSK3145095	RIPK1	None	Phase 2	-
	DNL747	RIPK1	None	Phase 1	-
	DNL104	RIPK1	None	Phase 1	-
	Ponatinib	RIPK3	None	Phase 2	-
	Dabrafenib	RIPK3	None	Phase 3	-
Pyroptosis	Emricasan	Caspases	None	Phase 2	-
	Disulfiram	GSDMD	None	Phase 2	-
	Tranilast	NLRP3	None	Phase 2	
	Dapansutrile	NLRP3	Phase 2	Phase 2	NCT04540120
	Pralnacasan	Caspase 1	None	Phase 2	-
NETosis	Sivelestat	Neutrophil elastase	None	Phase 3	-
	Disulfiram	GSDMD	Phase 2	Phase 4	[188]
	Dornase α	DNA	Phase 2	Phase 4	[189]

Therapeutic induction of neutrophil apoptosis at the site of inflammation may act to reduce aberrant inflammatory processes mediated by neutrophils in severe COVID-19. Neutrophils rely heavily on pro-survival BCL-2 family members for survival [190] and may be preferentially sacrificed with BH3 mimetic drugs that lower the threshold for the induction of intrinsic apoptosis. The rationale for killing activated neutrophils is supported by their involvement in producing pathogenic extracellular traps [67].

Local pathogen-induced inflammatory cytokine production such as TNF may provide an opportunity for preferential induction of apoptosis of infected lung epithelia and inflammatory immune cells such as macrophages. IAP inhibitor drugs, also known as SMAC mimetics, promote apoptotic cell death downstream of TNF receptor engagement. This approach could simultaneously facilitate rapid clearance of the pathogen while mitigating immune cell mediated inflammation.

**Figure 2.**
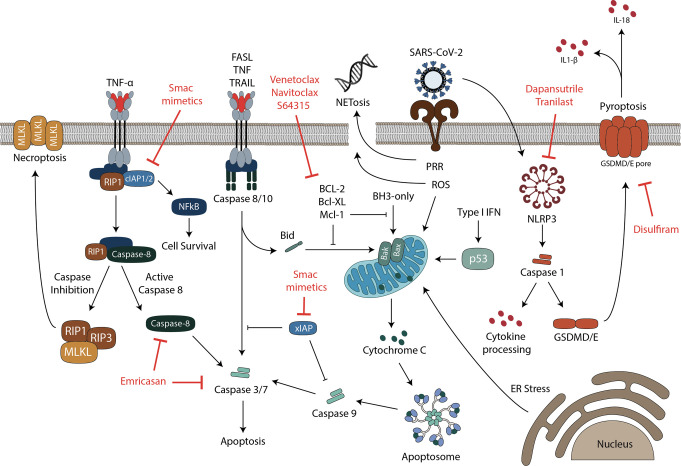
Cell death pathways involved in COVID-19 and potential therapeutic targets. Infection by SARS-CoV-2 was shown to trigger multiple cell death pathways in different cell types. Death can be induced in infected and uninfected cells, by both virus mediated and indirect mechanisms involving pro-inflammatory cytokines. Necroptotic cell death is induced by death receptor signaling, including TNF receptor 1 (TNFR1) or Toll-like receptors (TLR), a type of pattern recognition receptors (PRRs). Downstream of TNFR1, receptor interacting serine/threonine kinases 1 (RIPK1) regulates signaling leading to activation of NF-kB, apoptosis or necroptosis [155]. Necroptosis occurs when RIPK1 promotes the activation of RIPK3, while Caspase-8 is inhibited [155]. Activated RIPK3 then phosphorylates the pseudokinase mixed lineage kinase domain-like (MLKL), leading to its activation, oligomerization and translocation to the cell membrane where it forms pores and eventually leads to cell death [156,157]. Extrinsic apoptosis on the other hand, is triggered downstream of death receptor signaling, when Caspase-8 is active. The process is regulated by cellular inhibitor of apoptosis proteins (cIAPs), which prevent activation of Caspase-8 and instead cause the activation of NFκB, leading to the production of inflammatory cytokines. Diverse stress stimuli, such as reactive oxygen species (ROS) and unfolded protein responses (ER stress), can activate the mitochondrial intrinsic death pathway, involving BCL-2-associated protein (BAX) and BCL-2 homologous antagonist killer (BAK). These proteins cause mitochondrial outer membrane permeabilization (MOMP), which results in activation of the apoptosome and initiator Caspase-9, leading to apoptosis. Intracellular BH3-only proteins can trigger death via the mitochondria, through inhibition of pro-survival B-cell lymphoma-2 (BCL-2)-regulated proteins [96]. The tumor suppressor protein p53 can also trigger apoptosis through the mitochondria and has been implicated as a direct transcriptional target of type I interferons during viral infections [115]. Both intrinsic and extrinsic cell death pathways lead to the activation of executioner Caspases (Caspases-3 and -7), to generate a cascade of proteolytic events ultimately leading to cell death [97]. A cross-talk between extrinsic and intrinsic apoptosis is mediated by the protein Bid, which migrates to the mitochondria upon cleavage to induce permeabilization of the outer mitochondrial membrane [192]. Pyroptosis is a necrotic and inflammatory for of programmed cell death regulated by inflammasomes as a response to infections or specific host derived proteins and crystals [124,125]. The inflammatory Caspase-1, human Caspase-4 and Caspase-5, or mouse Caspase-11 coordinate the pyroptotic pathway upon inflammasome activation [123], mediating processing of IL-1β and IL-18 into their active forms, as well as cleavage of gasdermin D (GSDMD). GSDMD induces pyroptotic cell death and the release of pro-inflammatory cytokines [123,126] through plasma membrane pore formation. Neutrophil extracellular trap (NET) formation (NETosis) is another form of programmed cell death associated with COVID-19. NETosis is triggered by neutrophils to neutralize intruders through the release of chromatin structures containing histones and antimicrobial proteins [62]. Promising potential therapeutics targeting specific proteins in different cell death pathways are shown in red.

Caspases are cell death executioners, but they also function to orchestrate cytokine maturation and release and facilitate cross-talk between multiple cell death and inflammatory processes. This may make them prime targets for therapeutic interventions to ameliorate COVID-19 (summarized in Figure 2). For instance, inhibition of Caspase-1, which cleaves and matures interleukin 1 and 18, may reduce the severity of COVID-19. Caspase 9 inhibition may ameliorate severe disease by interfering with its apoptotic or inflammatory processes [105]. Inhibiting lytic cell death processes such as pyroptosis and necroptosis may also limit the release of counterproductive inflammatory mediators, and various PAMPs and DAMPs, and may prove effective at mitigating severe disease [191].

Pharmacological inhibition of cell death processes has downstream implications by dampening the effect of inflammatory mediators and warrants further pre-clinical and clinical investigation for the treatment of severe COVID-19. Given the backup mechanisms and the interconnectivity between the main PCD pathways, treatment combinations targeting multiple effectors could potentiate the effect of single drugs. Many therapeutics that target cell death and inflammatory pathways (Table 2) could be examined to define their efficacy in managing severe COVID-19.

## Conclusion

Since the emergence of SARS-CoV-2, huge progress has been made in understanding COVID-19. It is clearly a complex host-driven disease, in which severity and mortality are dictated by a dysregulated host immune response, rather than by direct virus mediated killing of cells.

It has become evident that cytokines are the main drivers of COVID-19 pathogenesis, but their processing and release is inextricably linked to cell death, upstream and downstream of their production. This impedes our ability to dissect the separate roles of cell death and their contribution to COVID-19. Clearly, more studies are required to better understand the chronology and hierarchy of events. Are cytokines triggering cell death, leading to cytokine release, inducing more cell death and tissue damage? Or are infected cells intrinsically dying leading to cytokine release? Which cell death pathways dominate and what are the initiators and what are the consequences of disease?

Beyond the relevance of individual cytokines and cell death pathways it is important to understand the roles of different cell types in COVID-19. There have been very few studies using pre-clinical animal models to dissect the role of diverse cell death pathways and diverse cell types using gene targeted animals. The use of human ACE2 (hACE2) transgenic mice reproduces many aspects of human disease and allows testing of potential treatments [193], but creates complexity in generating compound mutant animals. Mouse adapted strains of SARS-CoV-2 have been developed and allow infection of gene targeted animals [194].

Multiple therapeutic approaches targeting cytokines and cytokine receptors have been evaluated in pre-clinical studies and have or are entering clinical trials to assess efficacy in managing COVID-19. Targeting the host cell death machinery should also be explored because it is likely a contributor or an effector in COVID-19 pathogenies. The pandemic has provided many lessons and one of them is that therapeutics targeting host cell pathways may have tremendous merit not just for the management of COVID-19 but future pandemics.

## Abbreviations

ACE-2: angiotensin converting enzyme 2
APCs: antigen presenting cell subsets
BAK: BCL-2 homologous antagonist killer
BCL-2: B-cell lymphoma-2
GSDMD: gasdermin D
IFN: Interferon
MERS-CoV: Middle East respiratory syndrome–related coronavirus
MLKL: mixed lineage kinase domain-like
MOMP: mitochondrial outer membrane permeabilization
NLRP3: Nod-like receptor family pyrin domain-containing 3
ORFs: open reading frames
PBMCs: peripheral blood mononuclear cells
PCD: programmed cell death
PERK: protein kinase R-like endoplasmic reticulum kinase
PRRs: pathogen recognition receptors
RIPK1: receptor interacting serine/threonine kinases 1
ROS: reactive oxygen species
SARS: Severe Acute Respiratory Syndrome
SARS-CoV: severe acute respiratory syndrome coronavirus
ZBP1: Z-DNA binding protein 1
